# Endogenous BDNF augments NMDA receptor phosphorylation in the spinal cord via PLCγ, PKC, and PI3K/Akt pathways during colitis

**DOI:** 10.1186/s12974-015-0371-z

**Published:** 2015-08-20

**Authors:** Miao Liu, Jarren C Kay, Shanwei Shen, Li-Ya Qiao

**Affiliations:** Department of Physiology and Biophysics, Internal Medicine Gastroenterology, Virginia Commonwealth University School of Medicine, 1220 East Broad Street MMRB 5046, Richmond, VA 23298-0551 USA

**Keywords:** BDNF, NR1, Signal transduction, Spinal cord, Central sensitization

## Abstract

**Background:**

Spinal central sensitization is an important process in the generation and maintenance of visceral hypersensitivity. The release of brain-derived neurotrophic factor (BDNF) from the primary afferent neurons to the spinal cord contributes to spinal neuronal plasticity and increases neuronal activity and synaptic efficacy. The *N*-Methyl-D-aspartic acid (NMDA) receptor possesses ion channel properties, and its activity is modulated by phosphorylation of its subunits including the NMDA receptor 1 (NR1).

**Methods:**

Colonic inflammation was induced by a single dose of intracolonic instillation of tri-nitrobenzene sulfonic acid (TNBS). NR1 phosphorylation by BDNF in vivo and in culture was examined by western blot and immunohistochemistry. Signal transduction was studied by direct examination and use of specific inhibitors.

**Results:**

During colitis, the level of NR1 phospho-Ser^896^ was increased in the dorsal horn region of the L1 and S1 spinal cord; this increase was attenuated by injection of BDNF neutralizing antibody to colitic animals (36 μg/kg, intravenous (i.v.)) and was also reduced in BDNF^+/−^ rat treated with TNBS. Signal transduction examination showed that the extracellular signal-regulated kinase (ERK) activation was not involved in BDNF-induced NR1 phosphorylation. In contrast, the phosphatidylinositol 3-kinase (PI3K)/Akt pathway mediated BDNF-induced NR1 phosphorylation in vivo and in culture; this is an additional pathway to the phospholipase C-gamma (PLCγ) and the protein kinase C (PKC) that was widely considered to phosphorylate NR1 at Ser^896^. In spinal cord culture, the inhibitors to PLC (U73122), PKC (bisindolylmaleimide I), and PI3K (LY294002), but not MEK (PD98059) blocked BDNF-induced NR1 phosphorylation. In animals with colitis, treatment with LY294002 (50 μg/kg, i.v.) blocked the Akt activity as well as NR1 phosphorylation at Ser^896^ in the spinal cord.

**Conclusion:**

BDNF participates in colitis-induced spinal central sensitization by up-regulating NR1 phosphorylation at Ser^896^. The PI3K/Akt pathway, in addition to PLCγ and PKC, mediates BDNF action in the spinal cord during colitis.

**Electronic supplementary material:**

The online version of this article (doi:10.1186/s12974-015-0371-z) contains supplementary material, which is available to authorized users.

## Background

Spinal central sensitization is suggested to be one of the key mechanisms underlying visceral hypersensitivity in response to visceral irritation and/or inflammation in patients and experimental animal models [1–3]. Alterations in the expression and activity levels of neurochemical compounds and ion channels in the spinal dorsal horn underlie the molecular mechanisms of spinal central sensitization and is modulated by release of neurotransmitters from the primary sensory neurons located in the dorsal root ganglia (DRG) [4, 5]. Following colonic inflammation such as colitis induced by chemicals including tri-nitrobenzene sulfonic acid (TNBS), zymosan, acetic acid, mustard oil, or dextran sulfate sodium, there are increased levels of neurotrophins and neuropeptides in the DRG and the spinal cord resulting in visceral hypersensitivity [6–10]. As a result of zymosan-induced colitis, visceral hyperalgesia in rats is mediated, at least in part, by spinal *N*-methyl-D-aspartate (NMDA) receptor and is attenuated by intrathecal administration of the non-competitive NMDA receptor channel pharmacological blocker [7]. Peripheral tissue and nerve injuries recruit NMDA receptor at synapses within the pain-related spinal dorsal horn which is highly considered as an important process in spinal central sensitization under several physiologic and pathophysiologic conditions [11–16].

The NMDA receptor has an ionotropic property which regulates Ca^2+^ influx and Ca^2+^-dependent physiological effects thereby regulating neuronal activity and synaptic efficacy. NMDA receptor forms a heterotetramer composed of two NMDA receptor 1 (NR1) and two NR2 subunits [17]. NMDA receptor function may depend on phosphorylation of the subunits of the NMDA receptor [18, 19]. The NR1 subunit can be phosphorylated at Ser^890^ and Ser^896^ by protein kinase C (PKC) and at Ser^897^ by protein kinase A (PKA) [20, 21]. This is also true in animal models. Phosphorylation of Ser^897^ of the NR1 subunit in the spinal dorsal horn and spinothalamic tract neurons after intradermal injection of capsaicin in rats is mediated by the PKA pathway and is sensitive to PKA inhibitors [21]. Phosphorylation of Ser^896^ of the NR1 subunit in the spinal dorsal horn following noxious heat stimulation of the rat hind paw is mediated by activation of PKC [22]. In cystitis, both Ser^896^ and Ser^897^ sites of the NR1 subunit are phosphorylated in the spinal cord [5]. The Ser^897^ but not Ser^896^ phosphorylation in the spinal cord is regulated by calcitonin gene-related peptide (CGRP) [5]. We speculate that brain-derived neurotrophic factor (BDNF) may be involved in PKC-mediated NR1 Ser^896^ phosphorylation in the spinal cord during visceral inflammation.

BDNF is a member of nerve growth factor family. The release of BDNF from the neuron somata in the DRG to the spinal dorsal horn, where BDNF binds high affinity receptor TrkB, influences associative synaptic plasticity and increases synaptic efficacy by refinement of neural connectivity [9, 23, 24]. The involvement of BDNF in sensory hypersensitive is proved by a line of evidence and is well discussed in several animal models. In particular with colitis, systemic injection of BDNF neutralizing antibody reverses colonic hypersensitivity in response to colonic distention [25]. Intrathecal infusion of BDNF neutralizing antibody via mini pump also attenuates colitis-associated bladder hyperactivity [10]. In other animal models, the role of BDNF in mediating sensory sensitization is observed during peripheral inflammation-induced somatic pain [26, 27], cancer-induced bone pain [28], nerve injury, etc. [29–32]. Traditionally, three signaling pathways are activated by BDNF in neurons: activation of the Ras-extracellular signal-regulated kinase (ERK) cascade leads to gene transcription promoting cellular growth, the phosphatidylinositol 3-kinase (PI3K)/Akt pathway has anti-apoptotic function, and activation of phospholipase C-gamma (PLCγ) initiates Ca^2+^ release or influx via modulation of store activity or ion channels possibly through diacyl glycerol (DAG)-mediated PKC activation [33]. These pathways are recently recognized as essential components in sensory hypersensitivity in several animal models [34–38].

In culture, BDNF is able to modulate NMDA receptor in activity-dependent manner [39]. In the spinal cord, BDNF regulates the activity of the NR2B-containing NMDA receptor thereby participating in the development of neuropathic pain [40, 41]. The interrelationship of BDNF with the NR1 subunit of the NMDA receptor has also been reported showing that BDNF can regulate NR1 protein synthesis in vivo and in culture [28, 42]. Earlier studies demonstrate a role of BDNF in inducing NR1 phosphorylation on Ser^897^, the traditional PKA site, via the ERK and PKC pathways in isolated spinal dorsal horn [43, 44]. The present study is undertaken to examine the possibility of BDNF in regulating the NR1 subunit of the NMDA receptor by phosphorylating Ser^896^, the traditional PKC site, in vivo and in spinal culture, and explore the signal transduction in an animal model of colitis-induced visceral hypersensitivity.

## Experimental procedures

### Animals and reagents

Adult male rats (150–200 g) were purchased from Harlan Sprague Dawley, Inc. (Indianapolis, IN). All experimental protocols involving animal use were approved by the Institutional Animal Care and Use Committee at the Virginia Commonwealth University. Animal care was in accordance with the Association for Assessment and Accreditation of Laboratory Animal Care (AAALAC) and National Institutes of Health guidelines. All efforts were made to minimize the potential for animal pain, stress, or distress as well as to reduce the number of animals used. TNBS, β-actin antibody, and other chemicals used in this experiment were purchased from Sigma-Aldrich (St. Louis, MO). Antibody against p-NR1 Ser^896^ was from Santa Cruz Biotechnology (Santa Cruz, CA). Antibodies for p-Akt and total Akt were from Cell Signaling Technology (Danvers, MA). Secondary antibodies for western blot were from Pierce Biotechnology (Rockford, IL), and secondary antibodies for immunohistochemistry were from Molecular Probes (Eugene, OR).

### Induction of colonic inflammation

Colonic inflammation was induced in fasted rats under anesthesia (2 % isoflurane). TNBS solution (60 mg/mL in 50 % EtOH) at a dose of 90 mg/kg (1.5 mL TNBS solution per kg body weight) was instilled into the lumen of the distal colon through a syringe-attached polyethylene catheter via the rectum 6 cm proximal to the anus. Control animals received similar volume of 50 % EtOH enema. Euthanasia of animals was performed on day 3 or day 7 after TNBS treatment. To ensure exposure of the distal colon to TNBS, rats were held head down by lifting up the tail for 1 min. This treatment regimen caused significant inflammatory responses identified by histology [45] as well as the up-regulation of pro-inflammatory factor in the distal colon (Additional file 1: Supplemental data).

### Treatment of animals with antagonists

Animals received antagonists via intravenous (i.v.) injection. Antagonists used in this study were BDNF neutralizing antibody (36 μg/kg body weight) and the PI3K inhibitor LY 294002 (50 μg/kg body weight). A single dose of antagonist was injected. When animals were examined on day 3 post colitis induction, the antagonist was injected on the same day and post-TNBS treatment. When animals were examined on day 7 post colitis induction, the antagonist was injected on day 3 after colitis induction. This treatment design was customized by us through preliminary studies.

### Tissue collection

The L1 and S1 spinal cord segments were dissected out and used for western blot, immunostaining, or acute culture. For western blot, spinal segments were freshly minced and homogenized in T-PER buffer (Pierce Biotechnology, Rockford, IL) supplemented with protease and phosphatase inhibitors (Sigma). For immunohistochemistry, spinal segments were fixed with 4 % paraformaldehyde in 0.1 M PBS (pH = 7.4) followed by 20 % sucrose for cryoprotection. For acute culture, spinal segments were freshly dissected out from naïve animals, transversely sectioned at a thickness of 250 μm, and randomly divided into groups for treatment.

### Western blot

The protein extracts were subject to centrifugation at 20,200*g* for 10 min at 4 °C, and the supernatant was removed to a fresh tube. The protein concentration was determined using Bio-Rad DC protein assay kit. Proteins were then separated on a 10 % SDS-PAGE gel and transferred to a nitrocellulose membrane. The membrane was blocked with 5 % milk in Tris-buffered saline for 1 h and then incubated with a specific primary antibody followed by HRP-conjugated secondary antibody. For internal loading control, the same membrane was stripped and re-probed with anti-β-actin antiserum or antibody to a non-phosphorylated form of the protein examined. The concentrations of antibodies used were p-NR1: 1:1000; p-Akt and Akt: 1:1000; and β-actin: 1:3000. The bands were visualized by enhanced chemiluminescence (ECL). Densitometric quantification of the immunoreactive bands was performed using the software FluorChem 8800 (Alpha Innotech, San Leandro, CA).

### Immunohistochemistry

The spinal cord segments were sectioned transversely at a thickness of 30 μm and were immunostained by free-floating method. Generally, sections were incubated with blocking solution containing 5 % normal donkey serum (Jackson Immuno Research, West Grove, PA) in PBST (0.3 % Triton X-100 in 0.1 M PBS, pH 7.4) for 30 min followed by specific primary antibodies overnight at 4 °C. After rinsing (3 × 10 min with 0.1 M PBS), tissues were incubated with fluorescence-conjugated species-specific secondary antibody for 2 h at room temperature. Following washing, the sections were mounted to slides and coverslipped with Citifluor (Citifluor Ltd., London). The sections were then viewed and analyzed with a Zeiss AxioImage Z1 Apitome fluorescent microscope.

The analysis of the immunoreactivity at the dorsal horn were done as previously reported by converting fluorescent images to a grayscale that ranged in intensity from 0 (black) to 255 (white) for the purpose of densitometry [46]. The same number of standard sized rectangles was overlaid on the area of interest (i.e., superficial dorsal horn in this study) for each spinal section. Intensity measured within the rectangles was averaged as one point.

### Spinal cord culture

Spinal cord segments were acutely cultured for 4–6 h in cell culture wells containing Dulbecco’s modified Eagle’s medium (DMEM) supplemented with 200 units/mL penicillin, 200 mg/mL streptomycin, and 100 mg/mL gentamycin. BDNF (50 ng/mL) was added to the culture medium and incubated for designated time points. After incubation, tissues were collected for western blot analysis. All cultures were maintained in a 10 % CO_2_ environment at 37 °C.

### Statistical analysis

Comparison between control and experimental groups was made by using Kruskal-Wallis non-parametric one-way ANOVA. For in vivo experiments, 4–6 animals were used for each experimental group. For culture, 3–4 independent experiments were performed. Results were presented as mean ± SE. Differences between means at a level of *p* ≤ 0.05 were considered to be significant.

## Results

### Up-regulation of NR1 phosphorylation at Ser^896^ in spinal dorsal horn during colitis

**Fig. 1:**
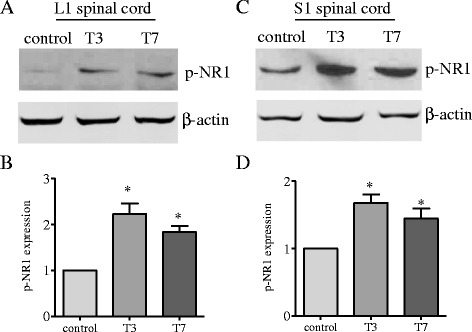
Up-regulation of NR1 phosphorylation in the L1 and S1 spinal cord during colitis. At 3 and 7 days post-TNBS treatment, the level of p-NR1 was examined by western blot (**a**, **c**) in the L1 (**a**, **b**) and S1 (**c**, **d**) spinal cord using a specific antibody against NR1 Ser^896^. Results showed that colitis caused significantly increases in the level of p-NR1 in the L1 and S1 spinal cord at both 3 days and 7 days post-TNBS treatment. *p < 0.05 vs. control

**Fig. 2:**
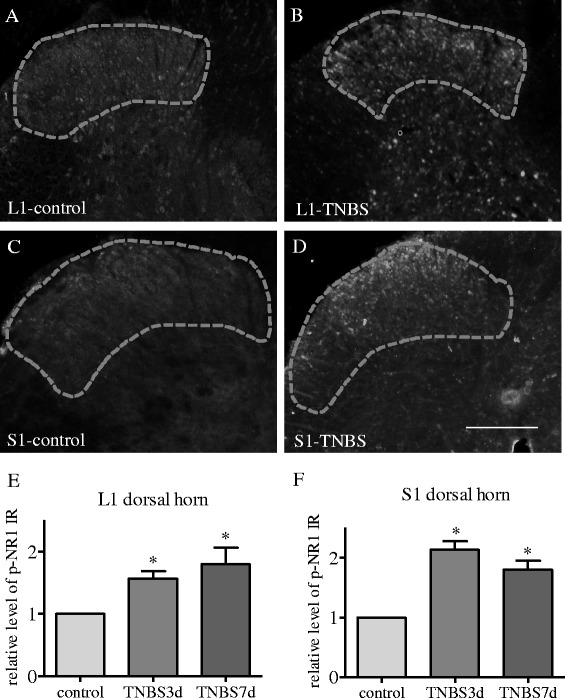
NR1 phosphorylation was increased in the dorsal horn of the spinal cord during colitis. Up-regulation of p-NR1 in the spinal cord during colitis was examined by immunohistochemistry (**a**–**d**). The p-NR1 immunoreactivity was increased in the region of the spinal dorsal horn in L1 (**a**–**b**) and S1 (**c**–**d**) spinal cord during colitis (compare **b** to **a** and **d** to **c**, circled area). Histograms (**e**, **f**) summarized the changes in the relative density of p-NR1 immunoreactivity in the L1 (**e**) and S1 (**f**) spinal dorsal horn at 3 days and 7 days of colitis. Microphotographs were from 3 days of colitis and control animals. *p < 0.05 vs. control. Bar = 250 μm

The expression level and the distribution pattern of the phospho (p)-NR1 in the spinal cord were examined by western blot and immunohistochemistry. We used specific antibody that recognized phospho-Ser^896^ on the NR1 subunit as described previously [5]. Western blot results showed that the level of p-NR1 Ser^896^ was increased in both L1 (Fig. 1a, b) and S1 (Fig. 1c, d) spinal cord at 3 days and 7 days post colitis induction. Immunohistochemistry staining showed that p-NR1 Ser^896^ immunoreactivity was expressed in several regions of the spinal cord. We paid particular attention to the dorsal horn region where the primary sensory neuron terminals ended. As shown in Fig. 2, colitis increased the immunodensity of p-NR1 Ser^896^ in the dorsal horn of the L1 (Fig. 2a, b) and S1 spinal cord (Fig. 2c, d). Summary data (Fig. 2e, f) presented the changes in p-NR1 Ser^896^ immunoreactivity in the L1 and S1 spinal dorsal horn at both 3 days and 7 days postcolitis. It is noteworthy that we have identified that phosphorylation of Ser^897^ of the NR1 subunit in the spinal cord was regulated by CGRP [5]. However, CGRP failed to regulate the phosphorylation of Ser^896^ on the NR1 subunit [5]. Thus, the present study focused on the examination of NR1 phosphorylation at Ser^896^ and aimed to identify factors that mediated NR1 Ser^896^ phosphorylation in the spinal cord in an animal model of colitis.

### BDNF regulated NR1 phosphorylation on Ser^896^ in vivo and in culture

**Fig. 3:**
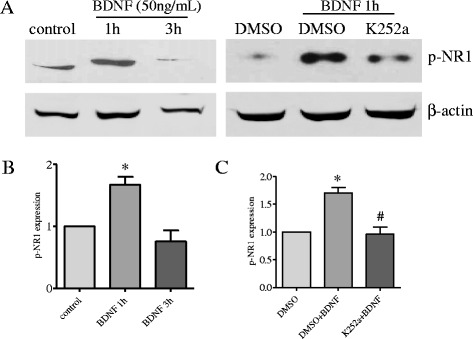
BDNF induced NR1 phosphorylation in the spinal cord which was reduced by K252a. Western blot (**a**) showed that incubation of the acutely cultured spinal cord slices with BDNF (50 ng/mL) elicited an increase in the phosphorylation level of NR1 at Ser^896^ (**b**). Pre-treatment of the spinal cord slices with a TrkB inhibitor K252a reduced BDNF-elicited NR1 phosphorylation (**c**). *p < 0.05 vs. control or DMSO alone; #*p* < 0.05 vs. BDNF (+DMSO)

**Fig. 4:**
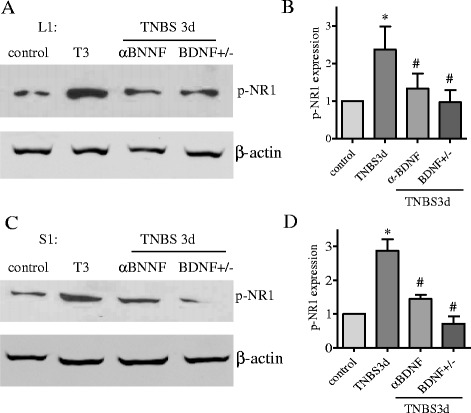
Up-regulation of NR1 phosphorylation in the spinal cord during colitis was regulated by endogenous BDNF. Western blot analysis of the L1 (**a**) and S1 (**c**) spinal cord from control rats, rats receiving TNBS or TNBS + BDNF antibody, and rats with partial deletion of BDNF (BDNF^+/−^) showed that blockade of BDNF action in vivo with BDNF neutralizing antibody or reduction of endogenous BDNF level by genetically engineering attenuated colitis-induced NR1 phosphorylation (**b**, **d**). *p < 0.05 vs. control; #*p* < 0.05 vs. TNBS

BDNF generated by primary afferent neurons is able to release to the axonal terminals upon stimulation. The primary afferent neurons that innervate the distal colon also project to the spinal cord dorsal horn due to their pseudounipolar nature with two split branches, one going to the periphery and another going to spinal cord. During colitis, the level of BDNF was elevated in the DRG [9], specifically in the colonic afferent neurons [47]. Thus, we examined whether BDNF was able to elicit NR1 phosphorylation at Ser^896^. Using an acute system of spinal cord culture as described previously [5, 9], we found that incubation of the spinal cord slices with BDNF increased the phosphorylation level of NR1 at Ser^896^, which was effectively blocked by pre-treatment of the spinal slices with Trk inhibitor K252a (100 nM, Calbiochem) (Fig. 3), suggesting that NR1 phosphorylation at Ser^896^ by BDNF was specific and was mediated by BDNF high affinity receptor. We then examined the role of endogenous BDNF on NR1 phosphorylation at Ser^896^. In both L1 and S1 spinal cord, neutralization of BDNF action in vivo with specific BDNF antibody reversed colitis-induced NR1 phosphorylation (Fig. 4). We confirmed these findings in BDNF^+/−^ rat (SAGE® Labs, Boyertown, PA) (Fig. 4).

### Signaling pathways in BDNF-induced NR1 phosphorylation in the spinal cord

**Fig. 5:**
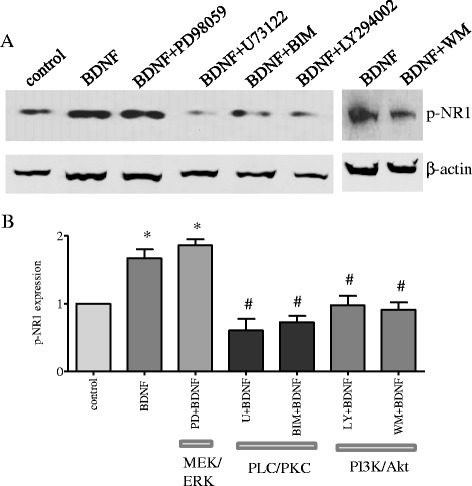
Signal transduction study of BDNF-induced NR1 phosphorylation in the spinal cord. The spinal cord slices were randomly separated into groups and pre-treated with a variety of specific inhibitors 30 min prior to BDNF stimulation. These inhibitors included PD98059 (*PD*) to block the MEK/ERK pathway, U73122 (*U*) to block the PLC pathway, bisindolylmaleimide I (*BIM*) to block PKC, LY294002 (*LY*), and wortmannin (*WM*) to block the PI3K/Akt pathway. Western blot (**a**) showed that PD had no effect on BDNF-induced NR1 phosphorylation while the rest of the inhibitors attenuated BDNF-elicited phosphorylation of NR1 at Ser^896^. Results were summarized in (**b**) indicating that the PLC/PKC and the PI3K/Akt, but not the MEK/ERK pathway was involved in BDNF-initiated NR1 phosphorylation. *p < 0.05 vs. control; #*p* < 0.05 vs. BDNF

We examined the involvement of three pathways, the MEK/ERK, PLCγ, and PI3K/Akt, in BDNF-induced NR1 phosphorylation at Ser^896^ in spinal slice culture. This was an acute system for quick initial screening of pathways. We used specific inhibitors: PD98059 to block MEK, U73122 to block PLCγ, and LY294002 to block PI3K. Since activation of PLCγ could facilitate DAG production leading to PKC activation, and NR1 Ser^896^ was considered to be phosphorylated by PKC [20], we also used bisindolylmaleimide I (BIM) to specifically block PKC in this system. Results (Fig. 5) showed that PD98059 (5 μM) did not have an effect on BDNF-induced NR1 phosphorylation; thus, the MEK/ERK pathway was not involved. The PLC inhibitor U73122 (10 μM) and PKC inhibitor BIM (1 μM) were able to attenuate BDNF-induced NR1 phosphorylation. The PI3K/Akt pathway was initially examined by using inhibitor LY294002 (5 μM) and then confirmed by another PI3K inhibitor wortmannin (0.5 μM). Both inhibitors blocked BDNF-induced NR1 phosphorylation at Ser^896^ (Fig. 5).

### NR1 phosphorylation at Ser^896^ in the spinal cord was mediated by the PI3K/Akt pathway in vivo in colitis

**Fig. 6:**
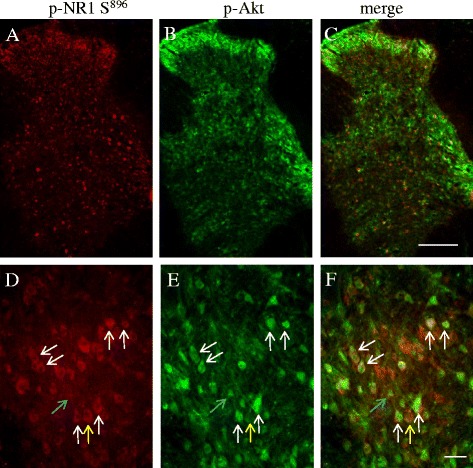
Co-localization of phospho-Akt and phospho-NR1 in the spinal cord. Double immunostaining of the spinal cord from TNBS animals showed that p-NR1 (**a**, **d**, *red cells*) and p-Akt (**b**, **e**, *green cells*) had similar anatomic distribution which included the dorsal horn and deep laminae (**a**–**c**). ApoTome scan revealed that majority of p-NR1 (**d**, *red cells*) and p-Akt (**e**, *green cells*) were expressed in the same cells (**f**, cells indicated by *white arrows*). Some of the p-NR1 positive cells did not express p-Akt (**d**–**f**, *yellow arrows*). Also some of the p-Akt positive cells did not express p-NR1 (**d**–**f**, *blue arrows*). Figure showed samples of the L1 spinal cord from animals at 7 days of colitis. Bar = 200 μm in **a**–**c**; 15 μm in **d**–**f**

**Fig. 7:**
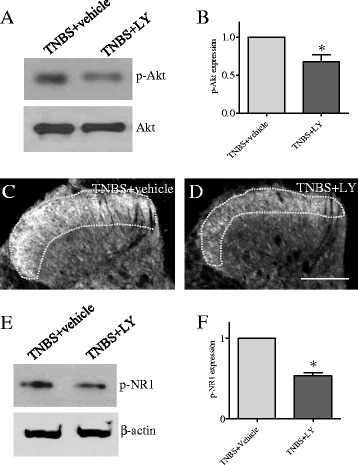
Inhibition of the PI3K in vivo with LY294002 blocked colitis-induced Akt activity and NR1 phosphorylation in the spinal cord. Animals receiving TNBS treatment (7 days) were divided into two groups: one receiving vehicle as control and one receiving LY294002 treatment. Animals treated with LY294002 had a reduced level of p-Akt in the spinal cord examined by western blot (**a**, **b**) and immunohistochemistry (**c**, **d**, *circled areas* are spinal dorsal horn), suggesting that inhibition of PI3K blocked Akt activity in vivo. Animals treated with LY294002 also had a reduced level of phospho-NR1 at Ser^896^ (**e**, **f**). Figure showed samples from S1 spinal cord. *p < 0.05. Bar = 250 μm

The involvement of the PI3K/Akt pathway in NR1 phosphorylation at Ser^896^ has not been reported previously. Thus, we confirmed our in vitro data (Fig. 5) with the in vivo system in animals induced for colitis. Double immunostaining (Fig. 6) showed that the p-NR1 (Fig. 6a, red stain) and p-Akt (Fig. 6b, green stain) had similar distribution pattern in the spinal cord (sections were L1 from TNBS 7 days). Higher magnification with ApoTome scan visualized the co-localization of p-NR1 and p-Akt in the spinal dorsal horn region (Fig. 6d–f, white arrows). These results suggested an association of Akt with NR1 phosphorylation in the spinal cord. We then injected the PI3K inhibitor LY294002 to colitic animals to block the PI3K/Akt activity in vivo (Fig. 7). We previously found that colitis increased the phosphorylation level of Akt in the L1 and S1 spinal cord at 7 days post-TNBS treatment [46]. Here, we found that LY294002 treatment reduced the Akt activity in the spinal cord during colitis examined by western blot (Fig. 7a, b showed S1 spinal cord; similar results were seen in L1 spinal cord, data not shown). Immunostaining showed that LY294002 treatment reduced p-Akt immunoreactivity in the spinal dorsal horn region during colitis (Fig. 7c, d showed L1 spinal cord; similar results were seen in S1 spinal cord, data not shown). LY294002 treatment also reduced colitis-induced NR1 phosphorylation at Ser^896^ (Fig. 7e, f).

## Discussion

Colitis-induced spinal central sensitization is attributable to posttranscriptional and posttranslational regulation in the spinal dorsal horn and is modulated by mediators generated in the primary afferent neurons. The NMDA receptor is one of the key molecules in regulating neuronal excitability and synaptic plasticity. The present study investigated the signaling pathways by which colonic inflammation facilitated phosphorylation of the NR1 subunit of the NMDA receptor in the spinal dorsal horn and identified that endogenous and exogenous BDNF were able to elicit NR1 phosphorylation at Ser^896^. In the spinal cord, colitis caused an increase in the level of NR1 Ser^896^ phosphorylation which was attenuated by inhibition of endogenous BDNF action with a specific BDNF antibody. The level of NR1 phospho-Ser^896^ was also reduced in BDNF^+/−^ rats treated with TNBS. The signaling pathways that transduced BDNF signal in the spinal cord leading to NR1 phosphorylation at Ser^896^ included the PLCγ, PKC, and the PI3K/Akt pathways. Even though the ERK pathway was also activated in the spinal cord by BDNF during colitis [9], the activation of this pathway did not lead to NR1 phosphorylation at Ser^896^. The phosphorylation of NR1 at Ser^896^ was traditionally recognized as the PKC site and was phosphorylated by PKC [20]. The present study revealed an additional pathway, i.e., the PI3K/Akt pathway, in mediating NR1 Ser^896^ phosphorylation in the spinal cord. The PI3K/Akt pathway was reported to mediate substance P (SP)-regulated NR2B subunit of the NMDA receptor [4], but had no role in CGRP-induced NR1 phosphorylation at Ser^897^ [5]. Taken together, these results suggest that inflammation-induced spinal central sensitization characterized by site- and isoform-specific phosphorylation of the NMDA receptor is regulated by primary afferent release of neuropeptides and neurotransmitters such as BDNF that is examined in the present study. Specific signal transduction pathways are involved.

Visceral hypersensitivity is a highly complex entity that can occur due to hyperexcitability of the primary sensory afferents and dysregulation of spinal neurons (central sensitization) that modulate nociceptive transmission. Following noxious stimulation (e.g., colitis in the present study), excitatory neurotransmitters such as BDNF, substance P, and CGRP release centrally from the primary afferent neurons into the spinal dorsal horn [46, 48–50] where they can bind to their respective receptors and facilitate signal transduction [4, 9, 35, 46], ultimately resulting in pain hypersensitivity (central sensitization) [51]. The mechanism and contribution of neuropeptides to central sensitization has been well discussed by Seybold [52]. These neuropeptides are often stored in large dense core vesicles of unmyelinated (C) and small myelinated (Aδ) terminals, and their release are triggered by higher firing frequency-evoked intracellular Ca^2+^ concentration and/or by persistent stimuli [53–56]. Neuropeptides sometimes also co-store with small molecule transmitters such as glutamate which is released from both the large and small myelinated fibers that can synapse on NMDA receptor-containing neurons in the spinal cord in regulation of nociception [57, 58].

The biological responses to neuropeptide release in the spinal cord involve a series of changes in the intracellular components. Activation of kinases is a key process in receptor-mediated signal transduction. The present study characterized three major pathways that transduce the BDNF signaling in neurons: the MEK/ERK pathway, the PI3K/Akt pathway, and the PLCγ pathway. Among these three pathways, MEK/ERK pathway is activated by BDNF in the spinal cord during colitis [9]; however, it is not involved in BDNF-induced NR1 phosphorylation at Ser^896^ characterized in the present study. We have previously reported that the phosphorylation level of Akt is increased in the L1 and S1 spinal cord during colitis [46]. In the present study, the PI3K/Akt pathway also mediates BDNF- and colitis-induced NR1 phosphorylation at Ser^896^ in the spinal cord. This is an additional pathway to the widely accepted NR1 Ser^896^ phosphorylation mechanism by which PKC is involved [20]. Akt is traditionally considered as a survival factor targeting Bcl proteins, pro-caspase, and Forkhead [59, 60]. Several lines of recent research have suggested a critical role of the PI3K/Akt pathway in mediating sensory sensitization in response to peripheral inflammation or injury [4, 5, 61]. The activity of Akt is regulated by PI3K-facilitated formation of phosphatidylinositol (3,4,5)-trisphosphate (PIP3) which results in Akt trafficking and activation [62]. In the periphery, Akt is activated in the sensory neurons and regulates pain perception by activating TRPV1 receptor [61, 63]. In the spinal cord, the Akt activity can be regulated by BDNF, CGRP, and SP in a PI3K-dependent manner [4, 5, 46]. In terms of visceral inflammation, activation of the PI3K/Akt pathway is unable to regulate NR1 phosphorylation at Ser^897^, but mediates NR1 phosphorylation at Ser^896^ (the present study) in the spinal cord, suggesting that the PI3K/Akt pathway is important but not the sole pathway in visceral inflammation-induced NMDA receptor activation and spinal central sensitization.

The interrelationship of Akt activation and NMDA receptor activity has been demonstrated in several systems. A study in a formalin-induced hyperalgesia model shows that inhibition of the PI3K/Akt pathway blocks peripheral inflammation-induced phosphorylation of NR2 subunit of the NMDA receptor in the spinal cord [4]. In cerebellar granule, cell culture activation of Akt is also able to mediate forskolin-induced phosphorylation of Ser^897^ in the NR1 subunit [64]. In inflammatory and neuropathic pain models, the NR1 phosphorylation is enhanced in the spinal cord [65, 66], and in these studies, the antibodies used for NR1 phosphorylation are selective for either the Ser^897^ site alone or both the Ser^897^ and Ser^896^ sites together but not Ser^896^ alone. In our current study, we use an antibody that recognizes specifically Ser^896^ of the NR1 and find that this site is phosphorylated in vivo during colitis. We have previously tested that CGRP is unable to increase NR1 phosphorylation levels at Ser^896^ [5]. The present study demonstrates that p-NR1 Ser^896^ is regulated by BDNF-induced PLCγ and PKC cascade, consistent to the established findings [20, 67]. The present study also reveals that BDNF- and colitis-induced NR1 phosphorylation at Ser^896^ is regulated by the PI3K/Akt pathway. However, there is no biochemical evidence to show whether the PI3K and/or Akt can use Ser^896^ of NR1 as a phosphorylation substrate. Akt serves as a convergent point in signaling network and is activated by growth factors and G-protein coupled receptors and also cross talks with other signaling pathways in cells and tissues. In the complex PI3K/Akt signaling network, the mammalian target of rapamycin complex can promote the stability and activity of PKC [68]. In spinal slice culture, it takes 1 h for BDNF to stimulate NR1 phosphorylation, which implies that complicated signaling cross talk may exist within the spinal cord. Akt can be activated by a number of cytokines that are discovered in the spinal cord, such as the pro-inflammatory cytokine tumor necrosis factor (TNF) that is released from spinal glial cells [34, 69]. The involvement of cytokines in persistent pain has been well discussed recently [70]. These studies along with our current findings suggest a key modulatory role of the PI3K/Akt pathway in the generation and maintenance of visceral and somatic hypersensitivity and pain by being activated by a number of inflammatory and neuronal factors and regulating a number of ion channels.

## Conclusion

The role of BDNF in mediating sensory hypersensitivity has been well recognized. The present study explores the molecular mechanisms and pathways that underlie the BDNF action in the spinal cord during colitis. BDNF in the spinal cord regulates NR1 phosphorylation which is mediated by specific signaling pathways. Especially, the PI3K/Akt pathway is found to be an additional pathway to PKC in regulating the NR1 phosphorylation at Ser^896^. The PI3K/Akt pathway is also important in the regulation of the NMDA receptor activity due to its ability in mediating NR2B subunit [4, 64]. The phosphorylation of these subunits is regulated specifically by diverse neuropeptides, such as BDNF, CGRP, and SP, converging on Akt and ultimately leads to NMDA receptor activation, which further mediates Ca^2+^ flux and modifies the strength and efficacy of synaptic transmission.

## Additional file

Additional file 1:
**Supplemental data.** (PDF 18 kb)

## Abbreviations

BDNF: brain-derived neurotrophic factor
BIM: bisindolylmaleimide
CGRP: calcitonin gene-related peptide
DRG: dorsal root ganglia
ERK: extracellular signal-regulated kinase
NMDA: *N*-Methyl-D-aspartic acid
NR1: NMDA receptor 1
PI3K: phosphatidylinositol 3-kinase
PKA: protein kinase A
PKC: protein kinase C
PLCγ: phospholipase C-gamma
TNBS: tri-nitrobenzene sulfonic acid
