# Supplementary material for: Endogenous BDNF augments NMDA receptor phosphorylation in the spinal cord via PLCγ, PKC, and PI3K/Akt pathways during colitis
Source: J Neuroinflammation. 2015 Aug 20;12:151. doi: 10.1186/s12974-015-0371-z (PMC4545933; doi:10.1186/s12974-015-0371-z)
Supplement: Additional file 1: — Supplemental data. (PDF 18 kb) [file 12974_2015_371_MOESM1_ESM.pdf]

Supplemental Data

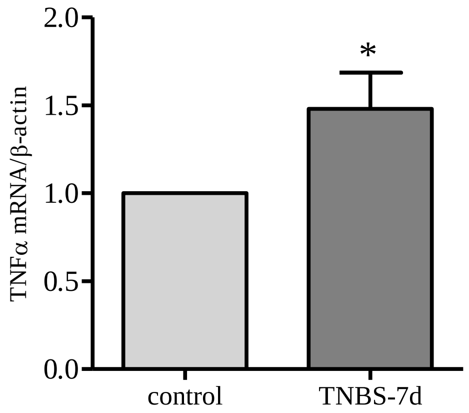

Supplemental data shows an increase in the expression level of pro-inflammatory factor tumor necrosis factor alpha in the distal colon during colitis. *, $p<0.05$. n=3.
